# Structural basis of inhibition of a transporter from *Staphylococcus aureus*, NorC, through a single-domain camelid antibody

**DOI:** 10.1038/s42003-021-02357-x

**Published:** 2021-07-05

**Authors:** Sushant Kumar, Arunabh Athreya, Ashutosh Gulati, Rahul Mony Nair, Ithayaraja Mahendran, Rakesh Ranjan, Aravind Penmatsa

**Affiliations:** 1 Molecular Biophysics Unit, Indian Institute of Science, Bangalore, India; 2 Principal Scientist, ICAR-National Research Centre of Camel (NRCC), Bikaner, India; 3 Present Address: Van Andel Institute, Grand Rapids, MI USA; 4 Present Address: Department of Biochemistry and Biophysics, Stockholm University, Stockholm, Sweden; 5 Present Address: Molecular Physiology and Biophysics, Vanderbilt University, Nashville, TN USA; 6 Present Address: Structural Parasitology Lab, International Centre for Genetic engineering and Biotechnology, New Delhi, India

**Keywords:** Antimicrobial resistance, X-ray crystallography

## Abstract

Transporters play vital roles in acquiring antimicrobial resistance among pathogenic bacteria. In this study, we report the X-ray structure of NorC, a 14-transmembrane major facilitator superfamily member that is implicated in fluoroquinolone resistance in drug-resistant *Staphylococcus aureus* strains, at a resolution of 3.6 Å. The NorC structure was determined in complex with a single-domain camelid antibody that interacts at the extracellular face of the transporter and stabilizes it in an outward-open conformation. The complementarity determining regions of the antibody enter and block solvent access to the interior of the vestibule, thereby inhibiting alternating-access. NorC specifically interacts with an organic cation, tetraphenylphosphonium, although it does not demonstrate an ability to transport it. The interaction is compromised in the presence of NorC-antibody complex, consequently establishing a strategy to detect and block NorC and related transporters through the use of single-domain camelid antibodies.

## Abstract (web summary)

Sushant Kumar et. al. report the 3.6 Å resolution crystal structure of NorC, a 14-transmembrane major facilitator superfamily member that is implicated in fluoroquinolone resistance in drug-resistant *Staphylococcus aureus* strains. The NorC structure was determined in complex with a single-domain camelid antibody that blocks access to the transporter and thus constitutes a new mode of inhibition.

## Introduction

Integral membrane transporters involved in multidrug efflux render pathogenic bacteria resistant to antimicrobial compounds through the reduced accumulation of drugs within cells^1^. Structurally and mechanistically diverse classes of primary and secondary active transporters facilitate the survival of pathogens against antibacterial compounds, either through direct efflux or through enhanced fitness or persistence^2,3^. The Gram-positive pathogen, *Staphylococcus aureus*, is known to cause mild skin and soft-tissue infections to severe infections like endocarditis, bacteremia, sepsis, and pneumonia^4^. A major factor that aids in the gain of multidrug resistance is the enhanced expression and activity of efflux transporters^5^. Among multidrug efflux transporters, the major facilitator superfamily (MFS) constitutes an extensive array of transporters with a promiscuous ability to transport a diverse array of substrates in both Gram-positive and Gram-negative pathogens^6–8^. Drug-resistant strains of *S. aureus* employ a diverse set of chromosomal and plasmid-encoded MFS transporters to gain antibiotic resistance^9^. Transporters like NorA, NorB, and NorC are chromosomally encoded and protect *S. aureus* against fluoroquinolones^9–11^. QacA and QacB are plasmid-encoded and provide resistance to monovalent and divalent quaternary ammonium compounds^12,13^.

MFS transporters involved in multidrug efflux primarily act by coupling efflux to proton gradients across the bacterial membrane^14^. The drug:H^+^ stoichiometry can differ, and substrate efflux can happen through electroneutral exchange or via electrogenic transport. MFS transporters are multi-pass integral membrane proteins comprising 12 or 14 transmembrane (TM) helices, and drug:H^+^ antiporters (DHA) are classified as DHA1 and DHA2 depending on the presence of 12 or 14 TM helices, respectively^6^. Multiple structures of DHA1 members, including MdfA, LmrP, and EmrD, have been solved in different conformational states that facilitate an understanding of the alternating-access in DHA members through the rocker-switch mechanism^15–17^. However, there is no representative structure for the DHA2 members that comprise well-studied transporters, including QacA/B, Tet38, NorB, and NorC. Besides *S. aureus*, numerous pathogens express DHA2 members to effectively counter antibacterial stress. Efflux transporters provide a potential therapeutic strategy to counter antimicrobial resistance through efflux pump inhibitors^18^. Blockers of antimicrobial efflux can enhance the efficacy of existing antibiotics, thereby serving as antibiotic adjuvants^19^.

In this study, we report the X-ray structure of NorC, a 14-TM putative efflux transporter representing a unique subset among MFS transporters in complex with a Zn^2+^-bound single-domain Indian camelid antibody (ICab). The structure of the ICab–NorC complex was solved at a resolution of 3.6 Å. Isolated initially as a crystallization chaperone for NorC, the ICab interacts with NorC through the insertion of CDR loops into the vestibule to effectively lock the transporter in an outward-open state. The transporter does not display a natural ability to transport fluoroquinolone or monovalent cations but can specifically interact with tetraphenylphosphonium, a monovalent cationic antibacterial. This interaction is blocked by the presence of the ICab. We anticipate that the structure of the DHA2 member, NorC, would facilitate the investigation of other DHA2 members in diverse pathogens, and the identification of single-domain antibodies that block efflux can be explored as a novel paradigm for efflux pump detection and inhibition.

## Results

**Fig. 1:**
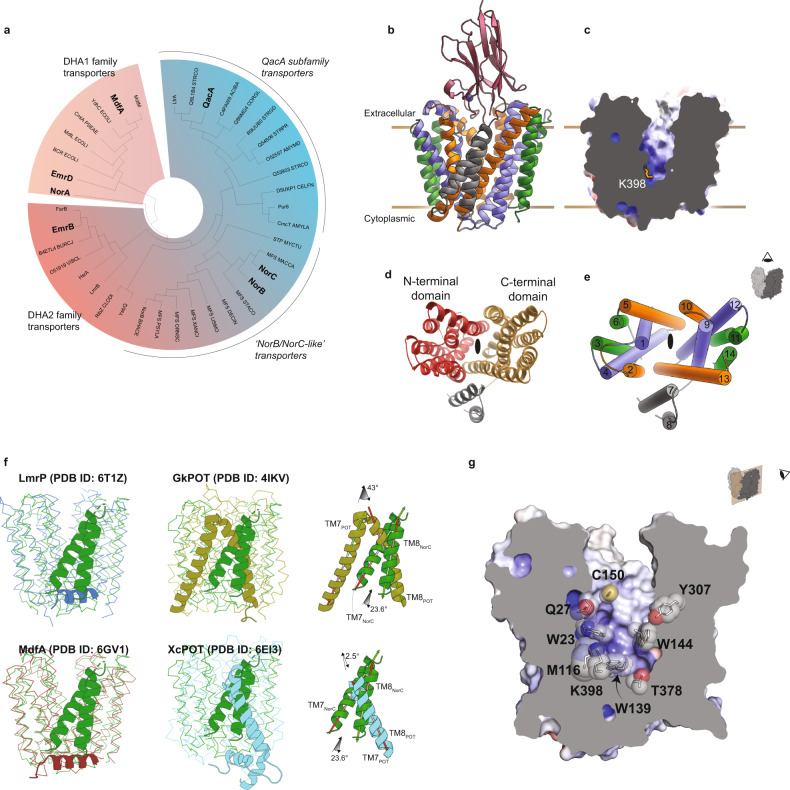
Phylogeny and X-ray structure of NorC. **a** Radial phenogram of DHA1 and DHA2 transporters. Characterized DHA2 transporters and their close homologs have been categorized with QacA/EmrB and their homologs for their sequentially coherent residues. **b** Side view of NorC bound to the ICab in an outward-open conformation. The transmembrane helices have been grouped in repeats of three helices to depict symmetric organization; vestibule lining TM helices 1, 4, 9, and 12 are in blue, 2, 5, 10, and 13 lining the domain interface in orange, 3, 6, 11, and 14 in green, and helices 7 and 8 are in gray. **c** APBS electrostatic representation showing positively charged region of the vestibule near the cytoplasmic end of NorC, owing to K398 (shown as sticks). **d**, **e** Note that TM7 and TM8 lie outside the primary pseudo-two-fold symmetric helical bundles that surround the vestibule. **f** Structural superposition of LmrP (blue, RMSD = 3.0 Å), MdfA (brown, RMSD = 2.5 Å), GkPOT (olive, RMSD = 4.4 Å) and XcPOT (cyan, RMSD = 4.2 Å) with NorC; angular differences between TMs 7 and 8 of NorC and those of POTs are shown in the same superposition. MdfA and LmrP are members of the DHA family while GkPOT and XcPOT are proton-dependent oligopeptide symporters from *Geobacillus kaustophilus* and *Xanthomonas campestris*, respectively. **g** Solid section of NorC showing vestibular environment.

NorC represents a unique subset among DHA2 members. Phylogenetic analysis reveals that NorC and related sequences form a separate clade among MFS transporters implicated in multidrug efflux (Fig.1a). Despite a clear prediction of 14-TM helices, NorC/NorB-like transporters differ substantially in comparison to the DHA2 members of the QacA-like transporters and the 14-TM proton-coupled oligopeptide symporters (POTs). Among the members of this subset, a high degree of sequence conservation is observed (~58–88%) (Supplementary Fig. S1). NorC and NorB display a high sequence identity of nearly 70%. It is observed that NorB can aid in the survival of *S. aureus* in an abscess environment and is also overexpressed in persister populations of antibiotic-resistant *S. aureus* strains^10,20,21^. Interestingly, the NorC/NorB-like transporters lack the typical protonation and conserved motif C residues characteristic of DHA^22^. While conventional DHA2 members retain one or more negatively charged residues for protonation-driven efflux, NorB and NorC lack negative charges facing the transport vestibule. For instance, D34 (TM1), a conserved acidic residue meant for substrate recognition and protonation among DHA2 members like QacA, is replaced by glutamine in the NorB/NorC clade. These changes may have significant consequences on the transport properties of NorC. We screened a wide array of potential substrates that could be transported by NorC, including fluoroquinolones. We employed capillary-based differential scanning fluorimetry (DSF) to identify compounds that can enhance the stability of NorC, thereby suggesting a potential substrate^23^. The stability increments were observed primarily for tetraphenylphosphonium (TPP^+^) (Supplementary Fig. S2). An analysis of the binding propensity for TPP^+^ revealed an ability to interact with NorC (described later). We also evaluated the ability of NorC to facilitate survival of an *E. coli* strain, deficient in *acrB*, *mdfA*, and *ydhE*, in the presence of antibacterial substrates like norfloxacin and tetraphenylphosphonium (TPP^+^) (Supplementary Fig. S3). The assay revealed no visible ability of NorC to protect against TPP^+^ and norfloxacin when overexpressed in *E. coli*.

### Crystallization and structure determination

The NorC WT (wild-type) protein was heterologously expressed and purified from *E. coli* membranes and crystallized in complex with a Zn^2+^-bound ICab that was identified and isolated in an earlier study^24^ (Supplementary Fig. S4). Despite obtaining single crystals, only a minor fraction would diffract and were susceptible to radiation damage. Multiple datasets were merged and scaled together to obtain a complete dataset to a resolution of 3.7 Å. The phases were estimated through Se-SAD phasing (described in methods). The resulting electron density was subjected to density modification, followed by manual model building and refinement. All the 14 TM helices could be modeled, and the main chain was traced with the help of selenium peaks resulting in the correct assignment of amino acids into the density. Multiple rounds of model building and refinement iteratively led to the convergence of refinement and generated a significantly improved electron density map (Supplementary Fig. S5). A mutation in TM13, K398A, allowed a marginal improvement of data quality to 3.6 Å with improved side-chain densities in the molecule leading to the refinement of the structure to acceptable R-factors (Table 1).

**Table 1:** Crystallographic data and refinement statistics.

*Data collection statistics*	NorC WT	NorC K398A
X-ray source	APS NE-CAT 24ID-C	APS NE-CAT 24ID-C
Temperature	100 K	100 K
Datasets merged^a^	8	6
Space group	*P*2_1_	*P*2_1_
Cell dimensions		
*a, b, c* (Å)	71.21, 139.87, 118.03	70.07, 139.14, 115.55
*α*, *β*, *γ* (°)	90, 106.03, 90	90, 105.7, 90
Wavelength (Å)	0.97910	0.97918
Resolution (Å)	49.18–3.65 (3.90–3.65)	139.45-3.60 (3.85–3.60)
Total number of observations	431,001 (68355)	426,838 (45048)
Unique reflections	24,592 (4248)	24,925 (4460)
Multiplicity	17.5 (16.1)	17.1 (10.1)
Data completeness	99.1 (95.2)	99.9 (99.3)
Mean *I*/*σI*	8.0 (1.0)	8.5 (1.1)
*R*_pim_ (%)	4.8 (105.7)	4.3 (87.9)
CC1/2	0.997 (0.563)	0.998 (0.486)
Anomalous completeness (%)	99.1 (95.2)	
Anomalous multiplicity	8.8 (8)	
DelAnomalous CC_1/2_	0.476 (0.022)	
*Refinement statistics*		
Resolution (Å)	48.96 (3.65)	111.68 (3.60)
Unique reflections	23409	23584
Rwork (%)/Rfree (%)	29.8/31.8	30.8/31.5
Non-hydrogen atoms/molecules		
Protein	7573	7487
Zn	2	2
Average B-factor (Å^2^)		
Protein	160.50	174.72
Zn	112.60	118.12
RMS bond lengths (Å)	0.014	0.015
RMS bond angles (°)	1.84	1.79
*Ramachandran plot*		
Favored (%)	91.61	90.97
Allowed (%)	8.39	9.03
Outliers (%)	0.00	0.00

^a^Data were obtained by scaling together multiple datasets that were collected on different crystals. The highest-resolution shell used in the final refinement is shown in parentheses.

### X-ray structure of NorC bound to single-domain camelid antibody

The structure of NorC retains a typical MFS fold and all the 14 TM helices of NorC could be modeled unambiguously in the electron density, although loop regions connecting TM6 with TM7 (189–198), TM7 with TM8 (223–226), and TM8 with TM9 (247–257) were missing due to inherent disorder (Fig. 1b, Supplementary Fig. S5). The TM helices are organized in two six-helix bundles, a conserved feature of MFS transporters, with the linker between the helical domains forming two additional TM helices leading to a 6 + 2 + 6 arrangement (Fig. 1d). The helices 1 to 6 and 9 to 14 are related by a pseudo twofold symmetry that facilitates conformational transitions through the rocker-switch mechanism^25^. Among the symmetrically arranged helices, TMs 1, 4, 9, 12 line the solvent-exposed vestibule forming cavity helices (blue), TMs 2, 5, 10, 13 are organized as rocker helices (orange) and 3, 6, 11, 14 are on the exterior facing the lipid interface forming the support helices (green) (Fig. 1e)^26^. The arrangement of the helical bundles is consistent with the organization in MFS transporter (MdfA and LmrP) structures in the outward-open state^15,16^. The MFS members, proton-coupled oligopeptide transporters (POTs) that are involved in the symport of di or tri-peptides, also comprise 14 TM helices^27–29^. However, all the POT structures known thus far were elucidated in the inward-open conformation unlike NorC, which is in the outward-open conformation. The relative positions of the additional TM helices 7 and 8 differ substantially in their orientation in NorC, in comparison to equivalent helices in POTs (Fig. 1f). Despite these differences, the presence of two additional helices as an insertion in the linker connecting the six-helix bundles among distantly related NorC and POTs makes this a consistent organization among 14-TM MFS members. The TMs 7 and 8, in NorC, are observed to occlude a wide opening between the helical bundles towards the outer leaflet of the membrane lined by TMs 2 and 13. When compared to the Hoechst bound LmrP structure^16^, the position of TMs 7 and 8 clashes with Hoechst, potentially alluding to the ability of TMs 7 and 8 to influence substrate interactions among NorC-like transporters (Supplementary Fig. S6).

**Fig. 2:**
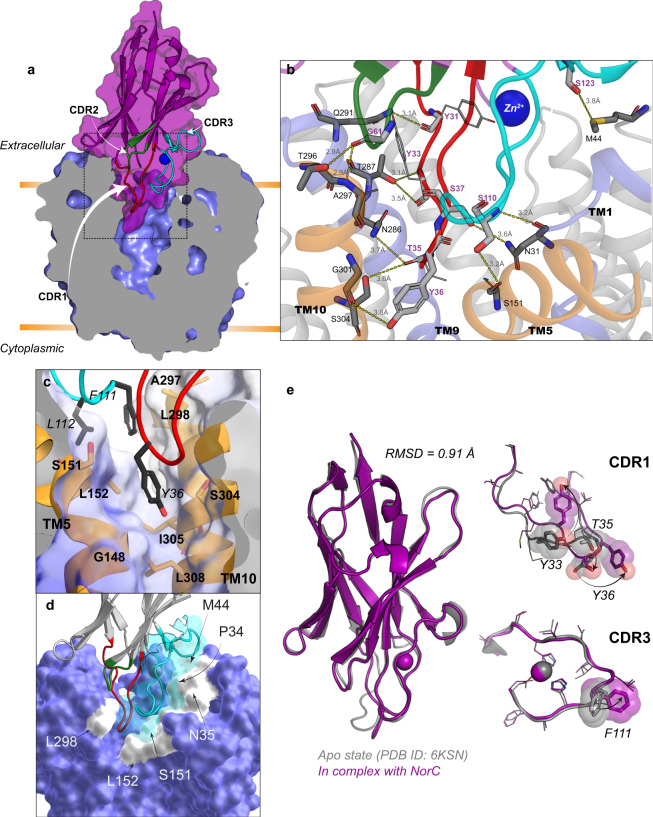
ICab interacts with NorC in an outward-open state. **a** Solid section of NorC (light blue) in complex with ICab (purple). CDRs 1–3 are shown in red, green, and cyan loops respectively, with Zn^2+^ as a blue sphere. The region of interest in dashed border is magnified in **b** with interchain H-bonds (dashed lines, lengths labeled) and participating residues (in sticks; lines shown for those not participating via their side chains, for clarity). NorC helices interacting with ICab through H-bonds are shown as cartoon. **c** Y36 (CDR1) and F111 and L112 (CDR3) involved in extensive hydrophobic interactions at the interface of the N- and C- terminal domain of NorC are shown with participating residues (sticks) and complementary NorC contour (transparent surface). **d** CDR3 (cyan cartoon and surface) forming a near-perfect shape complementarity with interacting residues from NorC (white surface patches) involved in polar and VdW interactions. **e** Structural alignment of ICab in native state (gray) and when bound to NorC (purple). CDR1 and CDR3 display the conformational changes that residues (sticks and transparent spheres) undergo during complexation (arrows).

The structure of NorC is solved in an outward-open conformation with entry into the vestibule blocked by the ICab that was used as a crystallization chaperone (Fig. 2a). Both aromatic and non-polar residues W23 (TM1), W139 (TM5), W144 (TM5), M116 (TM4) line the vestibule creating hydrophobic pockets as well as regions that can allow interactions with charged substrates (Fig. 1g). Unlike QacA, NorC lacks any negatively charged residues within the vestibule although a single cationic charge at K398 is observed in the cytosolic half of TM13 whose side chain is positioned facing the vestibule (Fig. 1c). Theoretical estimation of pKa of K398 yielded a value of 8.0^30^. This value is lower than the pKa of solvent-exposed lysine, which is usually around 10.4^31^. The reduction in pKa of lysine to a physiological range facilitates proton exchange during substrate transport. While acidic residues like glutamate and aspartate remain the predominant protonatable residues for substrate transport, lysines also couple protonation-driven transport as observed in Na^+^/H^+^ antiporters and ApcT family of amino acid transporters^32,33^. The location of K398, in NorC, coincides with the position of E407 in TM13 of QacA homology model where it was observed to be vital as both a general protonation site and substrate recognition site for certain substrates^34^. The vestibule also has a substitution of Q27 instead of acidic residues (D34) commonly observed in DHA members including QacA and MdfA that transport cations.

Most MFS transporters are identified by the presence of conserved motifs that are characteristic of the superfamily despite weak sequence identities. In NorC, the motif A with a consensus sequence of G_66_X_3_D_70_(K/R)XGR_74_X(K/R) is well-conserved and consistent with other MFS transporters (Supplementary Fig. S1). The D70 is involved in a salt bridge interaction with R74 of the same motif (Supplementary Fig. S7). R105 in TM4 forms the motif B and interacts with the main chain carbonyl groups of A26 (TM1) and occupies a similar position in comparison to its equivalent residue R112 in MdfA. The TM5 helix comprises motif C, which is characteristically present in antiporters and comprises a stretch of glycine residues interspersed with a GP dipeptide (GX_8_GX_3_GPX_2_GG)^22^. This motif is consistently present across numerous antiporter sequences and is absent in the case of symporters and uniporters^22^. While the glycine-rich sequence provides for the necessary freedom during conformational transitions from outward to inward-facing states, the main-chain carbonyl group of proline in the motif mediates interaction with the helices of the other domain via ordered water molecules in the inward-facing state, thus stabilizing that conformation, as seen in case of MdfA^15^. Incidentally, NorC retains a bulk of the glycine residues although the -GP- dipeptide is substituted by -CS- dipeptide (C150, S151) (Supplementary Fig. S1).

### ICab interacts with NorC in the outward-open state

The vestibule of NorC is open to the extracellular side although solvent access is limited through the interactions with ICab (Figs. 2a and 4a). Nanobodies derived from llamas have proven to be powerful tools in studying integral membrane protein structures^35^. While a large number of GPCR structures have been studied using nanobodies, a few transporters like LacY have been crystallized in complex with nanobodies that bind to extracellular face of the transporter^36^. Despite interacting with LacY with high affinity, the nanobodies allow the interaction of sugar in the binding pocket^36^. The ICab that we isolated has a longer CDR1 loop compared to other camelid and llama antibodies and the CDR3 loop harbors a unique Zn^2+^-binding site. Destabilizing the site leads to a loss of interactions with NorC^24^. The CDR1 loop has an additional -STYS- motif that forms a β-turn and inserts deep into the vestibule of NorC, to nearly half its depth (Fig. 2a) forming multiple polar and hydrophobic interactions with NorC (Fig. 2b). The motif wedges between symmetry-related helices TM5 from the N-terminal domain and TM9 and TM10 from the C-terminal domain. TM5 of MdfA undergoes angular shifts of 15° and a clockwise twist of 45° while changing from inward-open to outward-open conformation to facilitate alternating-access^15^. The TM5 of NorC is blocked by the presence of ICab whose CDR1 as well as CDR3 interacts with it. The presence of ICab CDR1 loop in the NorC vestibule further prevents the TM9 from curving as observed in the equivalent helix in MdfA(TM7) and restrains it as a linear helix (Supplementary Fig. S8). The CDR3 also interacts closely with residues in TMs 1, 2 and 5 towards the extracellular face (Fig. 2d), which undergo substantial shifts during the rocker-switch motion. While the epitope residues do not display any interaction with the Zn^2+^ ion directly, it is evident from the NorC-ICab complex that Zn^2+^-binding allows the CDR3 to have a conformation that facilitates high-affinity interactions with NorC. The residues F111 and L112 of ICab also wedge in the gap between TMs 5 and 10 that are part of the rocker helices (TMs 2, 5, 10, 13) that facilitate conformational changes in MFS transporters (Fig. 2c).

### NorC interaction induces conformational changes within ICab

**Fig. 3:**
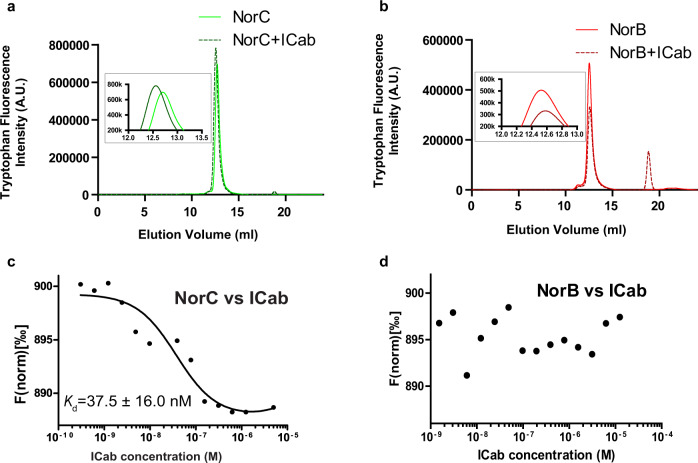
ICab specifically interacts with NorC. **a** Tryptophan fluorescence trace of NorC in FSEC, shifts to a slightly higher molecular mass when complexed with ICab. **b** NorB does not display a shift indicating a lack of interaction with ICab (inset). **c**, **d** Corresponding MST profiles of NorC and NorB respectively with *K*_d_ = 37.5 ± 16 nM for ICab and NorC but no detectable affinity for NorB. *n* = 2 for NorB vs ICab titration and *n* = 3 for NorC vs ICab for independent replicates. Source data are provided in the source data file.

The ICab used in this study displays a unique mode of interaction with the NorC. It has a high affinity of 38 nM and interacts specifically with NorC without any cross-reactivity to NorB, despite their close similarity, as observed in fluorescence-detection size-exclusion chromatography (FSEC) and microscale thermophoresis (MST) experiments (Fig. 3a, b). A comparison of the ICab crystal structures in the NorC-bound and free forms reveals subtle conformational changes in the CDR1 loop when it interacts with the antigen. The β-turn undergoes a straightening in its position and the T35 undergoes a 3.0 Å shift in its Cα position. Similarly, the Y36 in the β-turn undergoes a displacement of its phenol side chain by 3.3 Å to facilitate its wedging between TMs 5 and 10 (Fig. 2e). The CDR3 largely retains a conformation in the bound state similar to that of the free ICab with a minor displacement of about 1.5 Å in the region between 109 and 115. However, the side chain of F111 undergoes a massive shift in the χ1 torsion angle with 148° rotation to interact with NorC residues L152 and L298 (Supplementary movie SM1).

### ICab blocks access to antibacterial compounds

**Fig. 4:**
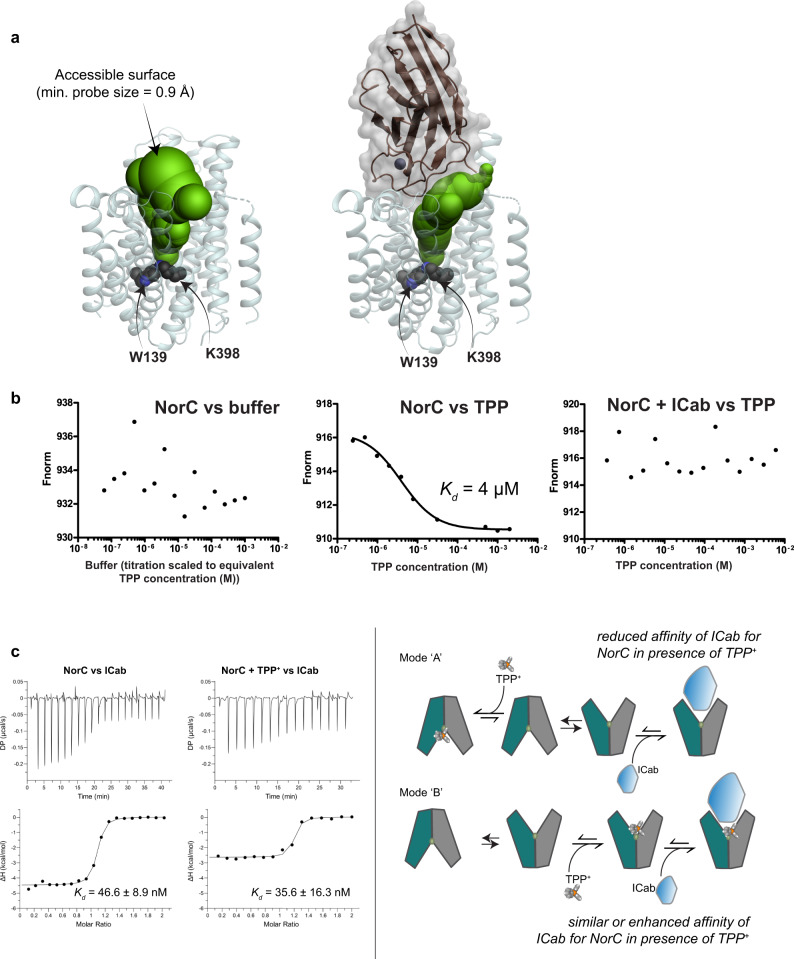
ICab interaction alters solvent accessibility of NorC vestibule. **a** Water accessible surface (green spheres) in NorC’s vestibule starting from W139/K398 (black spheres), in the absence (left) and presence (right) of ICab. The outward-open conformation completely blocks accessibility from the cytosolic side of NorC. **b** Microscale thermophoresis profiles for NorC and tetraphenylphosphonium (TPP^+^) in the presence and absence of ICab. *K*_d_ = 4 ± 0.8 μM for NorC and TPP^+^. *n* = 4 for NorC-TPP^+^ titration for independent replicates. The value provided is a concurrent representative from one. **c** ITC traces of ICab titrated against NorC WT in the presence and absence of TPP^+^ (offset corrected differential power, top panel; baseline subtracted binding isotherm with integrated peaks normalized against moles of injectant, bottom panel) concurs with mode “B” of TPP^+^ and ICab binding. *n* = 2 for independent replicates. Source data is provided in the source data file.

The ability of ICab to interact with the vestibule results in a substantial reduction of solvent accessibility within the vestibule of NorC, likely compromising substrate interactions (Fig. 4a). The natural substrates of NorB/NorC-like transporters are unknown. Most members related to NorC however retain an amidohydrolase in an open reading frame alongside the transporter gene in the di/tri-cistronic mRNA, which could be involved in processing the substrates transported by NorC. While we do not observe a direct efflux of fluoroquinolones with NorC (Supplementary Fig. S3b), we do however observe specific interactions with TPP^+^ with a *K*_d_ value of 4 μM despite lacking a propensity to transport cationic antibacterials (Fig. 4b). TPP^+^ interaction is observed in both NorB and NorC K398A leading to a nominal enhancement of stability (Δ*T*_*m*_ = +1.2 °C and +2.7 °C, respectively) (Supplementary Fig. S3a). This is entirely plausible in the context of other MFS transporter structures like LmrP, which has a detergent molecule, a phospholipid and a cationic dye (Hoechst) observed in distinct subsites within the vestibule^16^. We suggest that TPP^+^, given its multiple phenyl groups could stack and interact with the aromatic side chains within the NorC vestibule, thereby acting as an allosteric inhibitor of NorC instead of being a substrate. This presents two potential modes of TPP^+^ affecting ICab interactions with NorC. If TPP^+^ binding induces a conformational shift of NorC to occluded or inward-open states or binds competitively to the ICab binding site, the ICab interactions with NorC would weaken leading to reduced affinity (mode A, Fig. 4c). On the contrary, if TPP^+^ interacts with the outward-open state of the transporter and does not block ICab binding to the transporter the affinity would remain unaltered (mode B, Fig. 4c). We observe that while ICab binding blocks TPP^+^ interactions with NorC, the binding of TPP^+^ with NorC, on the contrary, does not influence the binding of ICab. In the presence of 1 mM TPP^+^, ICab interacts with similar affinity of 35.6 ± 16 nM with NorC (Fig. 4c). We could however observe a reduction in the enthalpy of ICab binding to NorC-TPP^+^ complex and suggest that TPP^+^ interaction likely pre-stabilizes a conformation favored for ICab binding (Fig. 4c, Supplementary Table ST1). However, the free energy of binding remains unaltered, hinting at the possibility that TPP^+^ too, like ICab, binds to the same conformation of NorC without impeding the interaction of the ICab.

### ICab and TPP^+^ binding induce a conformational shift of NorC

**Fig. 5:**
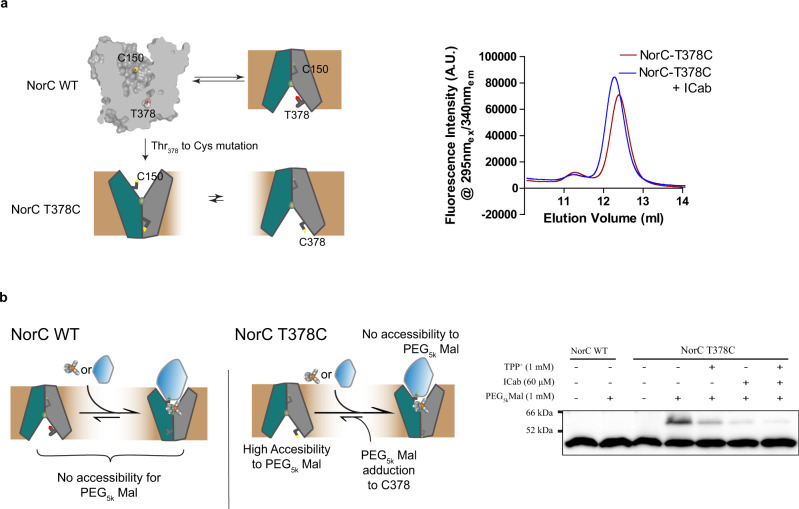
Characterizing NorC conformational shifts in response to ICab and TPP^+^ binding in *E. coli* membranes. **a** Schematic showing the accessibility of C150 and T378 of NorC in different conformations in native membranes. FSEC profile with detergent purified NorC T378C incubated with ICab shows that the mutation does not alter NorC’s ability to interact with ICab. **b** Methoxypolyethylene glycol maleimide (or PEG5k Mal; avg mol wt = 5 kDa) accessibility assay in NorC’s vestibule in *E. coli* membranes. PEG5k Mal does not react with NorC WT in native membranes or in detergent purified form, while it readily interacts with NorC T378C, as seen in the western blot (probed with Anti-Histag antibody), but the reactivity decreases in presence of excess ICab, TPP^+^ or both. *n* = 8 for independent replicates. Source data are provided in the source data file.

In order to evaluate if NorC is capable of conformational transitions in the membrane in response to ICab or TPP^+^ binding, we devised a biochemical strategy. A polyethylene glycol 5 K maleimide (PEG-Mal) was used as a crosslinker to form a covalent adduct with exposed cysteines in NorC and observed for a 5 kDa shift in the migration of NorC in overexpressed *E. coli* membranes through western blots. Despite having an exposed cysteine (C150) in the outward-open conformation of NorC, in the absence of bound ICab, we failed to observe any PEG-Mal crosslinking with NorC WT suggesting the lack of PEG-Mal exposure of C150 in NorC. In order to evaluate whether NorC is in a likely cytosol-facing conformation, we further engineered a cysteine at T378 position (TM12), which likely does not change the conformation of NorC as the mutant protein retains interactions with the ICab (Fig. 5a). Interestingly, the T378C mutant displays a clear PEG-Mal adduct formation suggesting that NorC in the membranes exists in an inward-open conformation. We further investigated the conformation by incubating the membranes with an excess of ICab and TPP^+^ and observed that in both cases the extent of PEG-Mal crosslink is visibly reduced (Fig. 5b, Supplementary Fig. S9). A combination of ICab and TPP^+^ displays the least extent of crosslinking suggesting that both the ICab and TPP^+^ force the conformation of NorC into an outward-open state (Fig. 5b).

## Discussion

NorC structure is the first representative structure of the DHA2 family and reveals the architecture of this distinct class of transporters within the MFS fold. While the true physiological substrate(s) of NorC is not evident, both NorC and its closely related homolog, NorB are observed to protect *S. aureus* against fluoroquinolones, which are broad-spectrum antibiotics. In this study, we have identified that TPP^+^, a cationic substrate of antibacterial efflux pumps, interacts with the outward-open conformation of NorC. TPP^+^ interacts despite mutating the protonatable lysine in the vestibule (K398) suggesting that the compound, given its multiple phenyl groups, could interact at an allosteric site stabilizing the outward-open conformation of NorC. This is very different from competitive binding displayed by TPP^+^ in antibacterial efflux transporters, MdfA and QacA, wherein substituting the primary protonation sites leads to a clear loss of TPP^+^ interaction and transport propensity^34,37^. In the presence of the ICab, entry into the vestibule of NorC is clearly blocked causing a complete loss of interactions of the antibacterial compound TPP^+^ with NorC (Fig. 4a, b). The ICab–NorC interactions happen through the insertion of the CDR loops deep into the vestibule of NorC with the transporter stabilized in an outward-open conformation. In doing so, the ICab–NorC interaction, resembles a “bottle-cork” that prevents antibacterial compound interactions and enforces a lock on the conformational changes within the transporter.

The ability to detect and block efflux transporters is proposed as a promising strategy to aid and improve the efficacy of existing antibiotics. The use of nanobodies to target plasma membrane primary and secondary active transporters for therapeutic outcomes has been conceptually proposed^38^. Nanobodies that antagonize purinergic receptor activity have been developed^39,40^. Nanobodies have also been developed as blockers of both primary and secondary active eukaryotic transporters including the Zn-transporting P-type ATPase^41^ and the vesicular glutamate transporter (VGlut)^42^. Nanobodies have been developed as blockers of bacterial transporters like the cyanocobalamine (Vitamin B12) transporter BtuF^43^ and used as chaperones to study the structure of a peptide-oligopeptide transporter-like DptA^29^ and NorC. The ICab characterized in this study provides a high-fidelity detection tool to observe the presence of NorC in *S. aureus* populations. Its ability to wedge deeply in the NorC vestibule and lock it in an outward-open conformation serves as a proof-of-principle to use ICabs or nanobodies as efflux pump inhibitors and a potential strategy to overcome antimicrobial resistance.

## Methods

### Statistics and reproducibility

All experiments described were performed in independent replicates (and technical replicates as well in the case of DSF screening). All comparative statistical analyses were done on complete sets of experiments. To ensure no variability arising due to batch-to-batch variations of proteins, statistical analyses for DSF experiments were performed within both technical replicates as well as independent replicates. However, no conflation on technical and biological variability has been performed.

### Sequence alignment and phylogenetic analysis

NorC (Uniprot ID A0A0E1ACG1) was used to search for closely related homologous sequences in a non-redundant sequence database. To search for more homologous sequences amongst the known DHA family members, a transporter classification database was used. The alignment was carried out using clustalw program with iterative HMM clustering. The output properly aligned the TMs of all the query proteins—a benchmark we used to check the alignment accuracy for this highly divergent dataset. The alignment was used further for functional and phylogenetic analysis. The evolutionary history was inferred by using the maximum likelihood method and JTT matrix-based model^44^. The tree with the highest log likelihood (−35519.34) is shown. Initial tree(s) for the heuristic search were obtained automatically by applying Neighbor–Join and BioNJ algorithms to a matrix of pairwise distances estimated using the JTT model, and then selecting the topology with a superior log-likelihood value. A discrete Gamma distribution was used to model evolutionary rate differences among sites (2 categories (*+G*, parameter = 3.0280)). This analysis involved 39 amino acid sequences. There were a total of 611 positions in the final dataset. Evolutionary analyses were conducted in MEGA X^45^.

### Survival assays

To check for phenotype (resistance against antibacterials), C41 cells (transformed with IPTG inducible *pET16b-norC* or *pET16b-norA)* and JD838 cells (transformed with L-arabinose inducible *pBAD-norC, pBAD-empty* or *pBAD-qacA*) were grown as primary culture overnight. A secondary culture was inoculated using this and split into two parts: one treated for induction of protein expression (using 0.2 mM IPTG or 0.05% (w/v) L-arabinose) and the other left untreated. The induction was done when the O.D.s_600nm_ reached 0.4 A.U. The cultures were further grown till all of them had an O.D._600nm_ between 1.5 and 2.0 A.U.; the cultures were then diluted accordingly to bring their O.D.s to 1.0 and spotted in tenfold serial dilutions on 1.5% (w/v) agar plate containing 2% (w/v) luria bertani (LB) broth and 100 μg/ml ampicillin with/without inducer in the media. Control plates containing none of the antibacterials but ampicillin were spotted similarly with/without inducer. The cells were allowed to grow overnight at 37 °C. These assays were replicated independently with *n* = 5.

### Cloning, expression, and purification of NorC

Full length *norC* gene was cloned into pET-16b vector between restriction sites NcoI and KpnI with 8X-His tag at its C-terminus. The *E. coli* C41(DE3) cells were transformed with the cloned vector containing an ampicillin resistance cassette. A primary culture was grown in LB-broth (HiMedia) for 10–12 h at 37 °C. Primary culture was used to inoculate large scale culture (2–3 l) that were grown at 37 °C until the OD_600_ reached 0.4–0.5 following which protein expression was induced with 0.2 mM isopropyl-β-D-1-thiogalactopyranoside (IPTG). The cells were further grown for 12–15 h at 20 °C. Cells were harvested by centrifugation and flash-frozen in liquid N_2_ for storage or processed immediately. The cell pellet was dissolved in HBS (20 mM Hepes pH 7.0, and 200 mM NaCl). The cell suspension was lysed at high pressure of about 800 bar using a homogenizer (GEA Niro Soavi PandaPlus 1000 Homogenizer) and spun at ~100,000 *g* for 1 h at 4 °C. The pelleted membranes were resuspended in HBS and 1 mM PMSF using a rotor-stator homogenizer, following which DDM (n-Dodecyl-β-D-maltopyranoside, Anatrace) was added to a final concentration of 20 mM. The solution was gently mixed for 2 h at 4 °C to extract NorC into micelles, followed by ultracentrifugation at 100,000 *g* for 1 h to remove the insoluble debris. The supernatant was incubated with Ni-NTA beads pre-equilibrated with buffer A (1 mM DDM in HBS) at 4 °C for 1 h. The solution containing beads was transferred into a gravity column (Bio-rad) and washed with 50 column volumes of buffer A having 30 mM imidazole. The protein was eluted in buffer A containing 300 mM imidazole and concentrated using a 30 kDa cut-off centrifugal filter (Amicon, Merck Millipore). It was further purified by size exclusion chromatography using Superdex S-200 increase 10/300 GL column (GE Healthcare) in HBS containing 4 mM n-Decyl-β-D-maltopyranoside (DM, Anatrace). The purity of the protein was analyzed by SDS-PAGE. The mutant NorC K398A protein was purified in a similar manner.

NorC WT was labeled with L-selenomethionine by growing *E. coli* C41(DE3) cells, transformed with NorC-containing vector, in M9 medium supplemented with 50 mg/L of L-selenomethionine. The purification of SeMet-labeled NorC WT was similar to that of the native protein.

### ICab generation and purification

Generation and purification of ICab were carried out in a manner described previously^24^. The ICab isolated through yeast-display screening was subcloned into the pET-22b vector. *E. coli* Rosetta cells were transformed with *a vhh*-containing vector. Large scale cultures were grown in LB broth at 37 °C until OD_600_ reached 0.6 and protein expression was induced by adding IPTG to a final concentration of 0.5 mM; cells were further grown at 37 °C for 5–6 h. The cells were harvested and the protein was extracted from inclusion bodies using urea denaturation followed by refolding. The refolding was done using step-wise dialysis at 4 °C. The protein was further purified by size-exclusion chromatography using Superdex S-75 10/300 GL column (GE Healthcare) in HBS. The purity and integrity of the protein were checked by SDS-PAGE and MALDI-TOF.

### Site-directed mutagenesis

To perform site-directed mutagenesis of NorC, primers were designed to amplify the entire plasmid carrying *norc* gene in a single step with desired point mutation. The amplicons were treated with Dpn1 at 37 °C for 2–3 h to digest the wild-type plasmid. *E. coli* Top10 cells were transformed with the mutant amplicon and plated on an LB-agar plate containing 100 µg/ml ampicillin and grown overnight at 37 °C. The positive clones were confirmed by DNA sequencing. The mutant proteins were purified in a manner similar to that of the wild type protein.

### Fluorescence-detection size exclusion chromatography (FSEC)

FSEC was done using a high-performance liquid chromatography system attached to an autosampler and a multi-wavelength fluorescence detector (Shimadzu). In total, 10 μM each of NorC WT, NorC-K398A and NorB purified in DM buffer were incubated with 1:1.2 molar ratios of ICab and shifts in their elutions (measured at λ_ex_ = 295 nm, λ_em_ = 340 nm) in the same DM buffer were used to qualitatively determine whether or not ICab binds to them. A Superdex 200 increase 10/300 GL column was used for analyzing elution times.

### Crystallization and structure determination

Purified NorC WT (native and SeMet-derivative) and NorC K398A (native only) proteins were concentrated to 3 mg/ml using a 50 kDa cut-off centrifugal filter (Amicon, Merck Millipore) and mixed with ICab in a molar ration of 1:1.2. The crystallization was carried out by hanging drop vapor diffusion method at 20 °C where protein and crystallization conditions were mixed in a ratio of 3:2 v/v. NorC WT and NorC-K398A crystallized in a condition containing 0.1 M MES pH 6.0, 50 mM NaCl, 35.7 % PEG 600, 57 mM MgCl_2_, 10 mM YCl_3_ and 6.0 mM CHAPSO. The crystals appeared within 3–4 days but took about 2 weeks to attain full size. The crystals were washed in the crystallization condition and flash-frozen in liquid N_2_. The X-ray diffraction datasets were collected at the advanced photon source (APS) synchrotron, USA. The crystals diffracted X-rays to a resolution of about 3.6–4 Å.

All the data sets were indexed and integrated with XDS^46^. Eight data sets were merged and scaled for SeMet-derivative NorC WT using AIMLESS^47^ resulting in the highest resolution of 3.65 Å. The space group was determined to be P2_1_ with unit cell dimensions of *a* = 71.2 Å, *b* = 139.9 Å, *c* = 118.0 Å and *α* = *γ* = 90° and *β* = 106° (Table 1). The Matthews coefficient suggested two NorC-ICab complexes in the asymmetric unit with a solvent content of about 70%. SAD phasing was carried out using CRANK2^48^ in CCP4i2^49^. For the substructure determination, the highest resolution was set at 5.0 Å as the anomalous signal was very weak at a higher resolution. SHELX C and D^50^ in CRANK2 were used for substructure determination and refinement. The resulting phases were extended to a resolution of 3.65 Å and the electron density was subjected to density modification using PARROT^51^. Manual model building was carried out in the density modified map using Coot^52^. Selenium positions were used as markers to trace the main chain and place amino acids.

Multiple X-ray diffraction data sets were collected for the native NorCK398A crystals. Six best data sets were merged and scaled using AIMLESS to a resolution of 3.6 Å. The structure was determined by molecular replacement using PHASER^53^ with NorC WT structure as a model. The refinement of both the structures was carried out using REFMAC^54^ until the refinement converged. The resulting structures were validated using MOLPROBITY^55^. The data collection and refinement statistics are presented in Table 1.

### Structural analysis and visualization

All structural analysis and visual representations of atomic coordinates were done using the PyMOL Molecular Graphics System, Version 2.0 Schrödinger, LLC. The pKa of K398 was determined on the PROPKA server used through its extension in PyMOL(TM) 2.4.0^30^.

### Microscale thermophoresis for protein ligand interaction quantitation

Quantification of NorC’s interaction with TPP^+^ was carried out using microscale thermophoresis (Nanotemper)^56^. The protein was labeled with red Tris-NTA dye (Nanotemper) by mixing both in an equimolar proportion. The labeled protein was mixed with TPP^+^ such that the concentration of the labeled protein and TPP^+^ were 10 nM and 0.1 mM, respectively. A total of 16 twofold serial dilutions of TPP^+^ were prepared to keep the protein concentration constant at 10 nM. The experiment was carried out with Monolith™ NT.115 MST premium-coated capillaries. For NorC-ICab complex titrations against TPP^+^, 10 nM of NorC was mixed with ICab in a 1:3 molar ratio and the starting concentration of TPP^+^ was kept at 0.1 mM. For ICab titrations against NorC, 10 nM of NorC was used with a starting concentration of 5 µM for the ICab. The curves were fitted with the single-site binding model. To assess the same with NorB and ICab, identical concentrations were taken, with NorB in place of NorC.

### Differential scanning fluorimetry for substrate screening

The propensity of TPP^+^ to interact with NorC WT, NorB WT, and NorC-K398A was assessed using differential scanning fluorimetry (DSF) using Prometheus NT.48 instrument (NanoTemper Technologies)^57^. A temperature scan was carried out between 20 and 80–90 °C with a scan rate of 1 °C/min. All measurements were carried out at least in duplicates. The first derivative of the ratio of *F*_*e*mission_350 nm/330 nm was plotted against temperature, and shift in *T*_*m*_ was used to determine whether or not TPP^+^ binds to the proteins. Similar protocol was employed for initial substrate screening where 5 μM of NorC WT or NorC K398A was taken with a 1:10 molar ratio of each substrate and assessed on a temperature range of 20–80 °C. *n* ≥ 4 for every substrate tested.

### Isothermal titration calorimetry

The binding affinity of NorC with ICab was determined by ITC using Microcal PEAQ-ITC (Malvern Panalytical). Both NorC and ICab were purified in a buffer containing 20 mM HEPES, pH 7.0, 200 mM NaCl, and 4 mM n-decyl-β-D-maltopyranoside detergent. The titration consisted of 20 injections of 4 s duration each, with the first injection of 0.4 µl and all the subsequent injections of 2 µl. The time between two consecutive injections was kept at 120 s, and the sample in the cell was stirred at 750 rpm during the entire run. For the run involving TPP^+^, each injection volume was increased to 2.5 µl to accommodate a low signal-to-noise ratio, while the number of injections were reduced to 16 keeping the overall volume unchanged. The concentration of NorC in the cell was kept at 20 µM and the concentration of ICab in the syringe was kept at 200 µM. The titration was carried out at a constant temperature of 25 °C. For blank, ICab was titrated against buffer, and the heat of dilution was subtracted and offset corrected from the titration data against NorC. All the data were fit using Microcal PEAQ-ITC analysis software with one set of sites model^24^.

### PEG maleimide accessibility assay

*E. coli* JD838 cells were transformed with *pBAD norc-wt* and *pBAD norc-t378c* vectors and grown in 2% (w/v) LB broth overnight. These cells were used to overexpress NorC WT and its T378C mutant, lysed, and the membranes were isolated, using the protocol described earlier. Each of the membrane fractions was resuspended in 2 ml of phosphate buffer saline, pH 7.4, snap-frozen in Liq. N_2_ and stored in −80 °C in 30–50 μL aliquots for further use. For the PEG_5k_ maleimide adduction reaction, the 5 μl of each membrane was diluted in HEPES buffer saline (HBS, pH 7.0, containing 20 mM HEPES and 200 mM NaCl) 10×. In a 20 μl reaction, 5 μl of the diluted membrane was added. In total, 60 μM ICab and/or 1 mM TPP^+^ was added in some reaction mixes prior to the addition of 1 mM PEG_5k_ mal. The final mix was incubated for 10 min in dark at 25 °C before quenching the reaction with 100 mM β-mercaptoethanol. These membranes were solubilized in 3% SDS and centrifuged at 17,000 *g* for 30 min before loading the supernatant on a 10% resolving SDS-PAGE. The gel was then western transferred onto PVDF membrane and probed with 1:4000 of anti-His_8_ tag mouse primary antibody (Invitrogen) and 1:10000 HRP tagged goat anti-mouse secondary antibody (Invitrogen) and analyzed for band shifts after the adduction reactions. The blots were independently repeated eight times with multiple batches of membranes. A similar protocol was also used for SEC purified proteins, with 10 mM PEG_5k_ mal was used for the assay.

### Reporting summary

Further information on research design is available in the Nature Research Reporting Summary linked to this article.

## Supplementary information

Peer Review File

Supplementary Information

Description of Additional Supplementary Files

Supplementary Movie 1

Supplementary Data 1

Reporting Summary

## Data Availability

Data supporting the manuscript would be available upon reasonable request from the corresponding author. A reporting summary is available as a Supplementary Information file. The atomic coordinates and structure factors of NorC WT and NorC-K398A are deposited in the Protein Data Bank with PDB IDs 7D5P (10.2210/pdb7D5P/pdb) and 7D5Q (10.2210/pdb7D5Q/pdb), respectively. Source data are provided with this manuscript.
