# Supplementary material for: Structural basis of inhibition of a transporter from Staphylococcus aureus, NorC, through a single-domain camelid antibody
Source: Commun Biol. 2021 Jul 5;4:836. doi: 10.1038/s42003-021-02357-x (PMC8257674; doi:10.1038/s42003-021-02357-x)
Supplement: Supplementary file 2 — Supplementary Information [file 42003_2021_2357_MOESM2_ESM.pdf]

Structural basis of inhibition of a transporter from *Staphylococcus aureus*, NorC, through a single-domain camelid antibody

Sushant Kumar, Arunabh Athreya, Ashutosh Gulati, Rahul Mony Nair, Ithayaraja Mahendran, Rakesh Ranjan and Aravind Penmatsa*

Supplementary Information

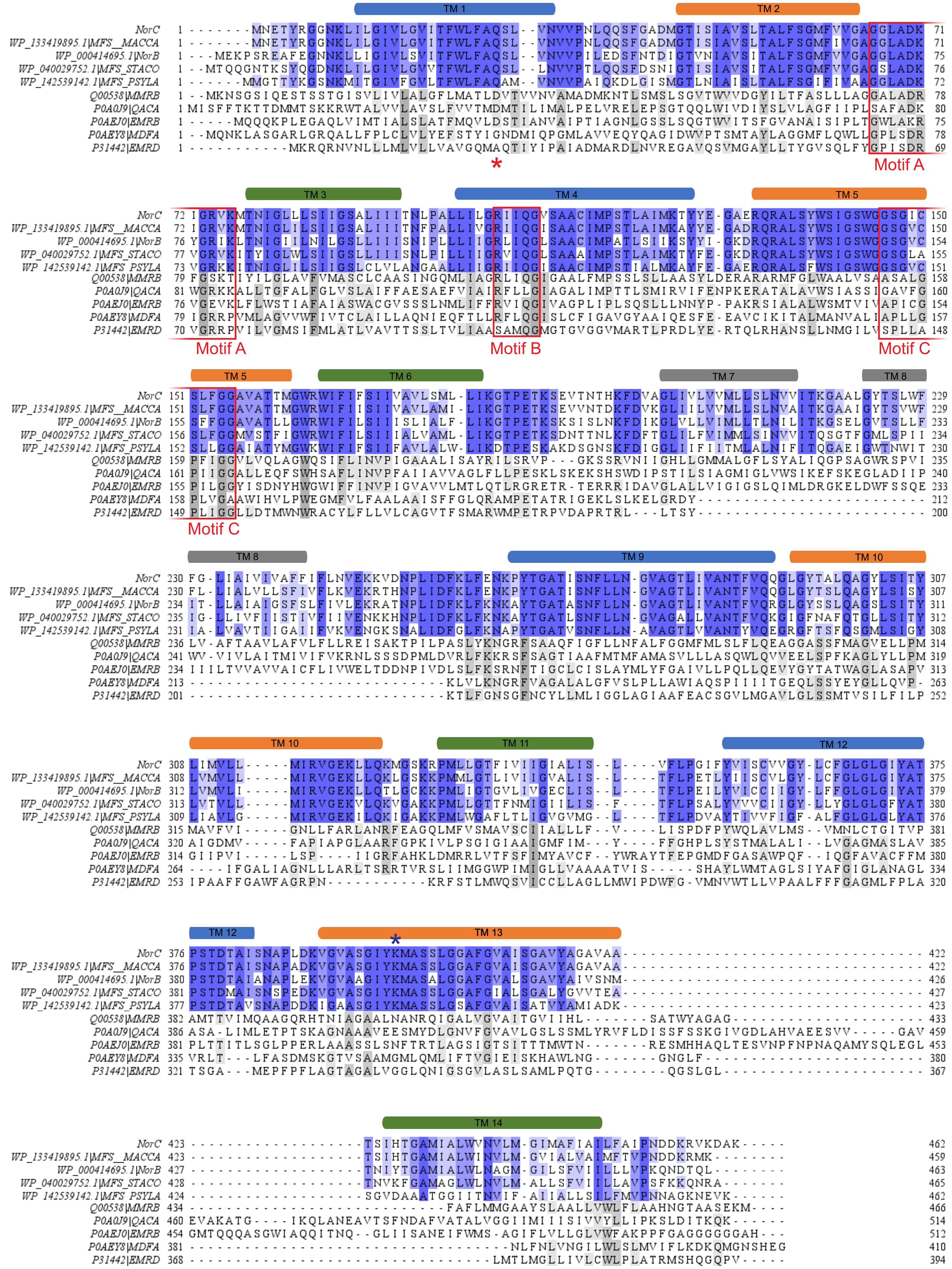

Supplementary Figure S1 | Sequence alignment of NorB/NorC-like and conventional DHA1 and DHA2 family members. Conservation is shown in blues and greys, respectively. Residue conservation declines as highlights go lighter. TM helices are identified as cylinders above the sequences. Motifs A, B and C are boxed in red. Asp34 (DHA1 and DHA2), and Lys398 (NorC/NorB subfamily) are marked with asterisks.

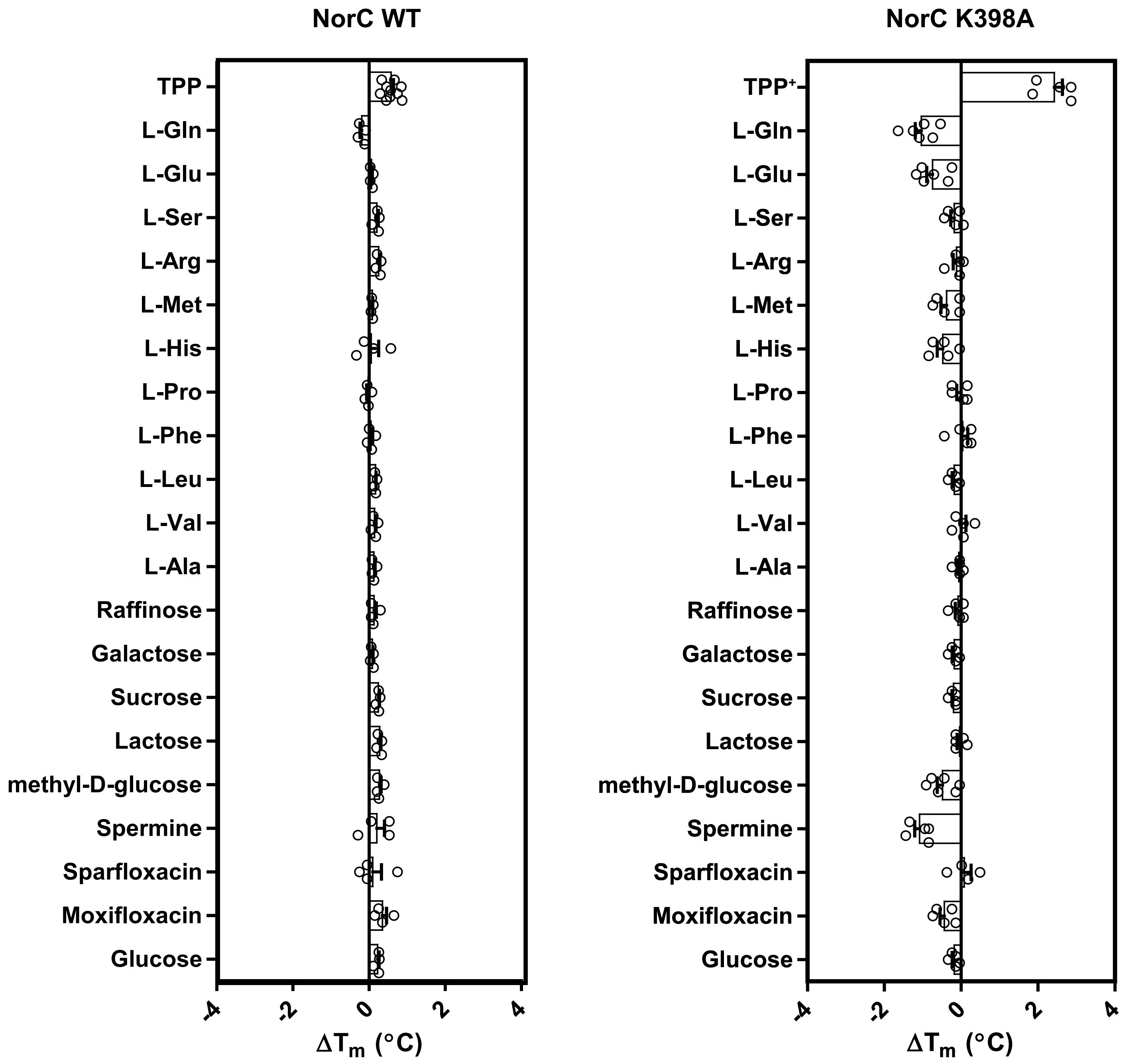

Supplementary Figure S2| DSF Screening of various compounds to find potential substrates of NorC. ΔT_m were calculated by subtracting T_m calculated for apo-protein for each experiment. n>4 for every substrate tested. Error bars indicate standard error of mean. Source data are provided in the source data file.

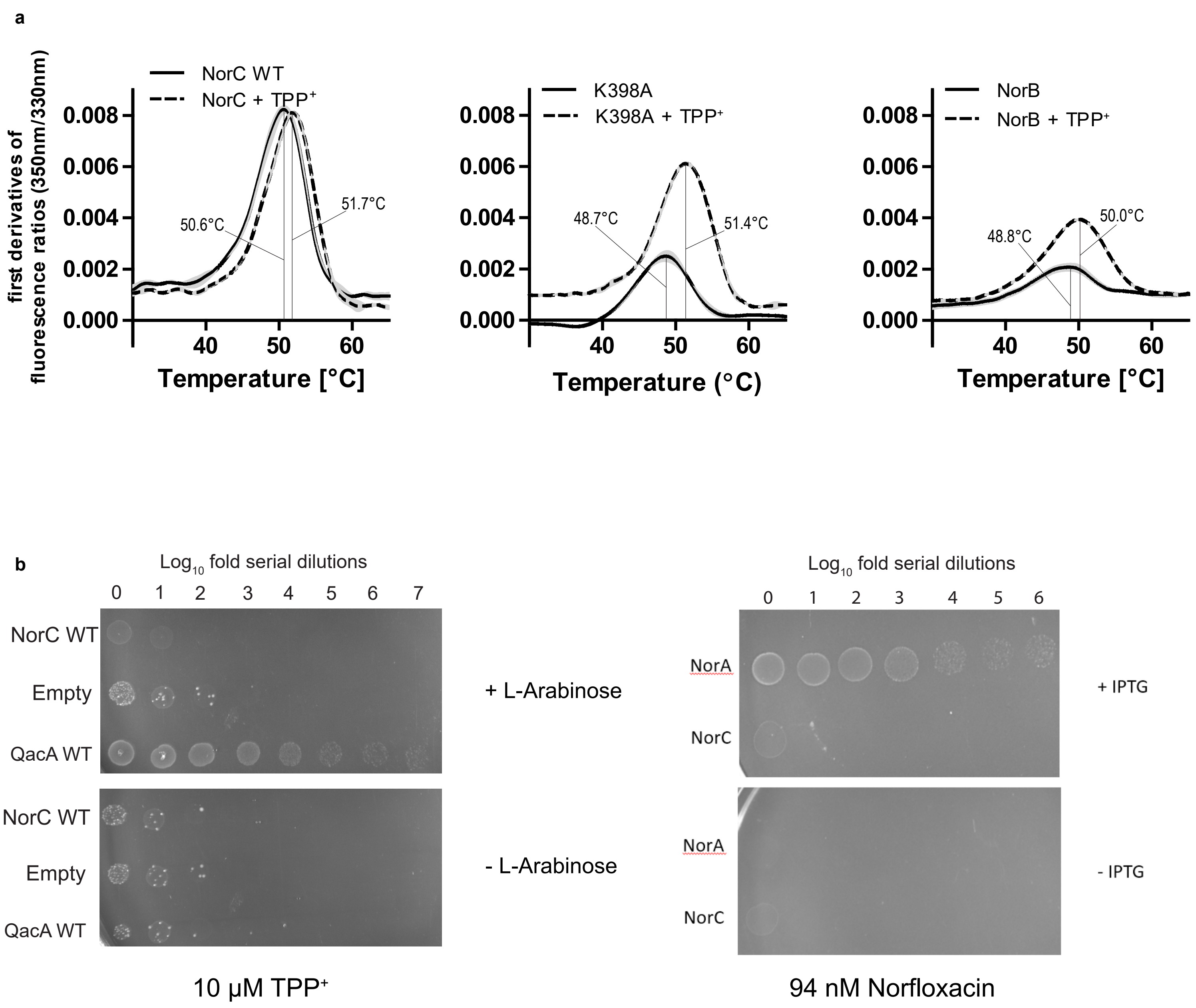

Supplementary Figure S3 | a. Differential scanning fluorimetric profiles of NorC WT, NorC K398A and NorB WT in the presence and absence of TPP^+; error bars are shown as grey shadows (n=2 for technical replicates). **b.** Survival assays done in the presence of tetraphenylphosphonium (TPP^+, using L-arabinose inducible pBAD24 expression vector) and norfloxacin (using IPTG inducible pET16b expression vector) at known MIC_{50}; n=4 for independent replicates. Source data are provided in the source data file.

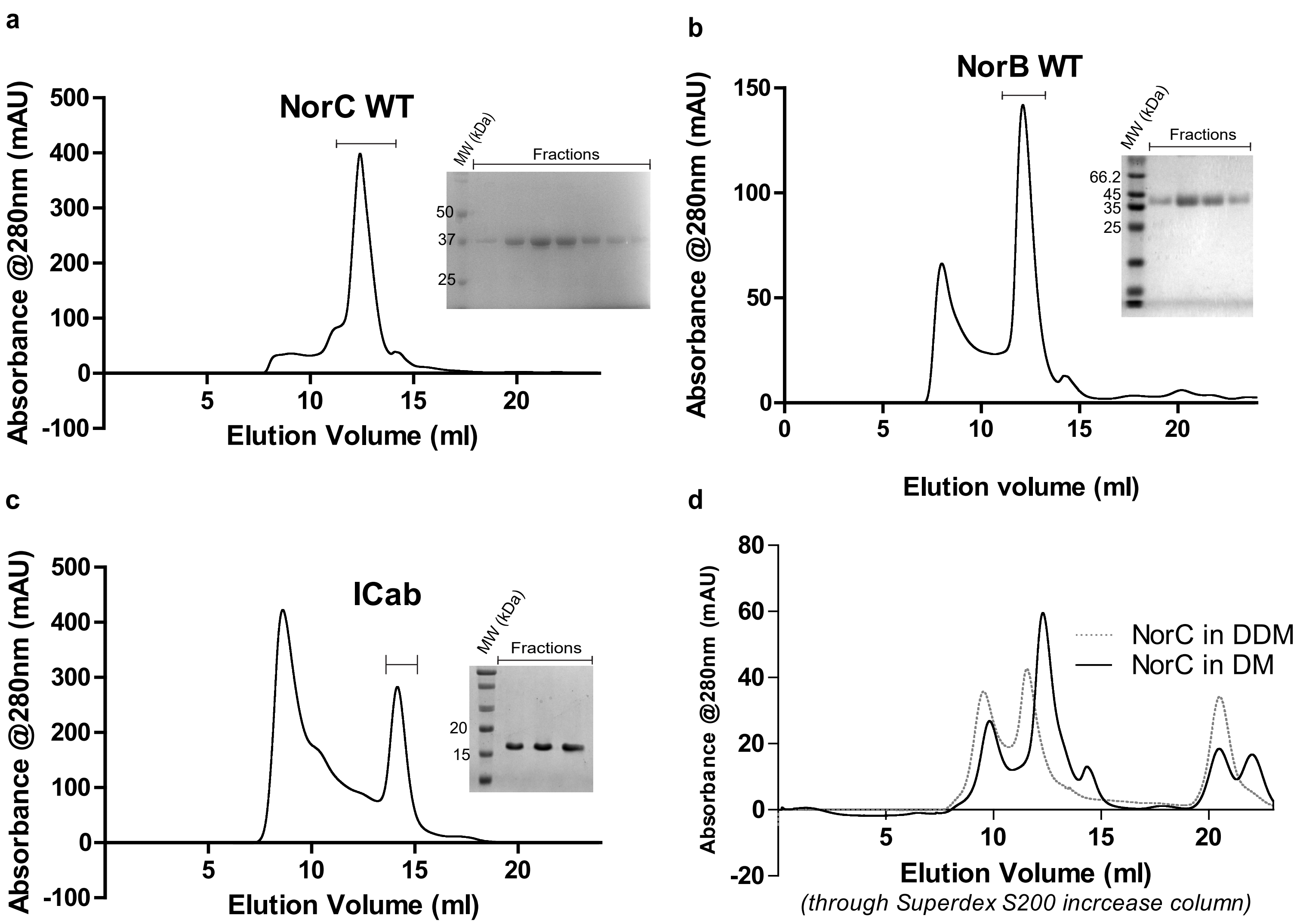

Supplementary Figure S4 | Size exclusion chromatography profiles and SDS-PAGE (insets) with corresponding fractions of **a.** NorC WT, **b.** NorB WT and **c.** ICab. **d.** Comparison of NorC WT size exclusion chromatography profiles in 1mM DDM (n-dodecyl-β-D-maltopyranoside, grey dotted trace) and in 4mM DM (n-decyl-β-D-maltopyranoside, black trace). Source data are provided in the source data file.

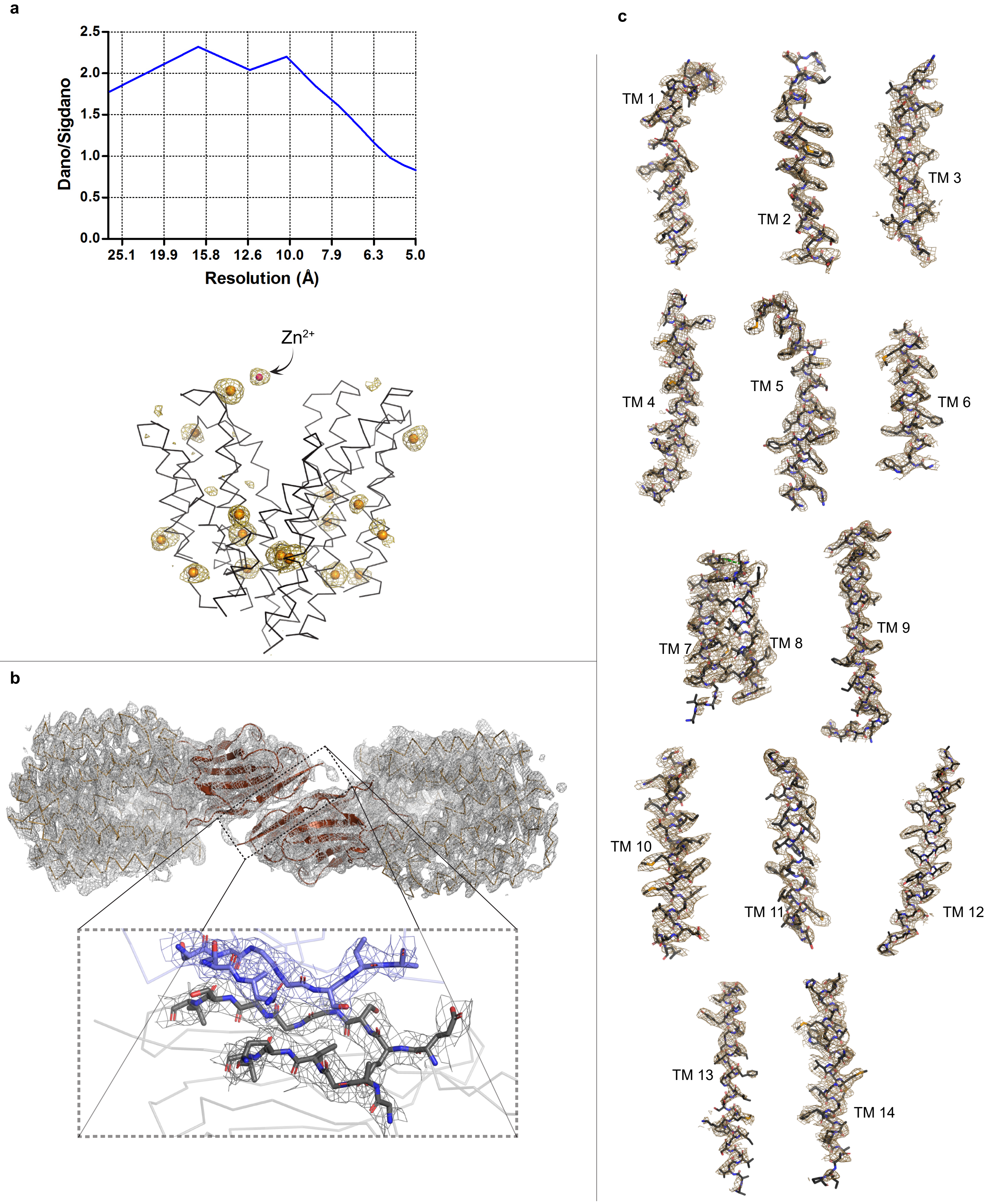

Supplementary Figure S5 | a. *(Top)* signal quality represented by $\Delta I_{Ano}/\sigma(\Delta I_{Ano})$. Anomalous phasing and substructure determination was done at 5 Å; *(bottom)* 16 out 17 Se atom positions were determined with their anomalous densities shown in golden spheres and meshes respectively. Zn^{2+} is shown in pink sphere. **b.** ASU contains 2 molecules of NorC and ICab each, shown with 2Fo-Fc map contoured at 1.0 σ; focused region of interest displays interactions between ICab chains (stick representation). **c.** Individual TM helices and their surrounding 2Fo-Fc map at 1.0 σ carved at 2.0 Å.

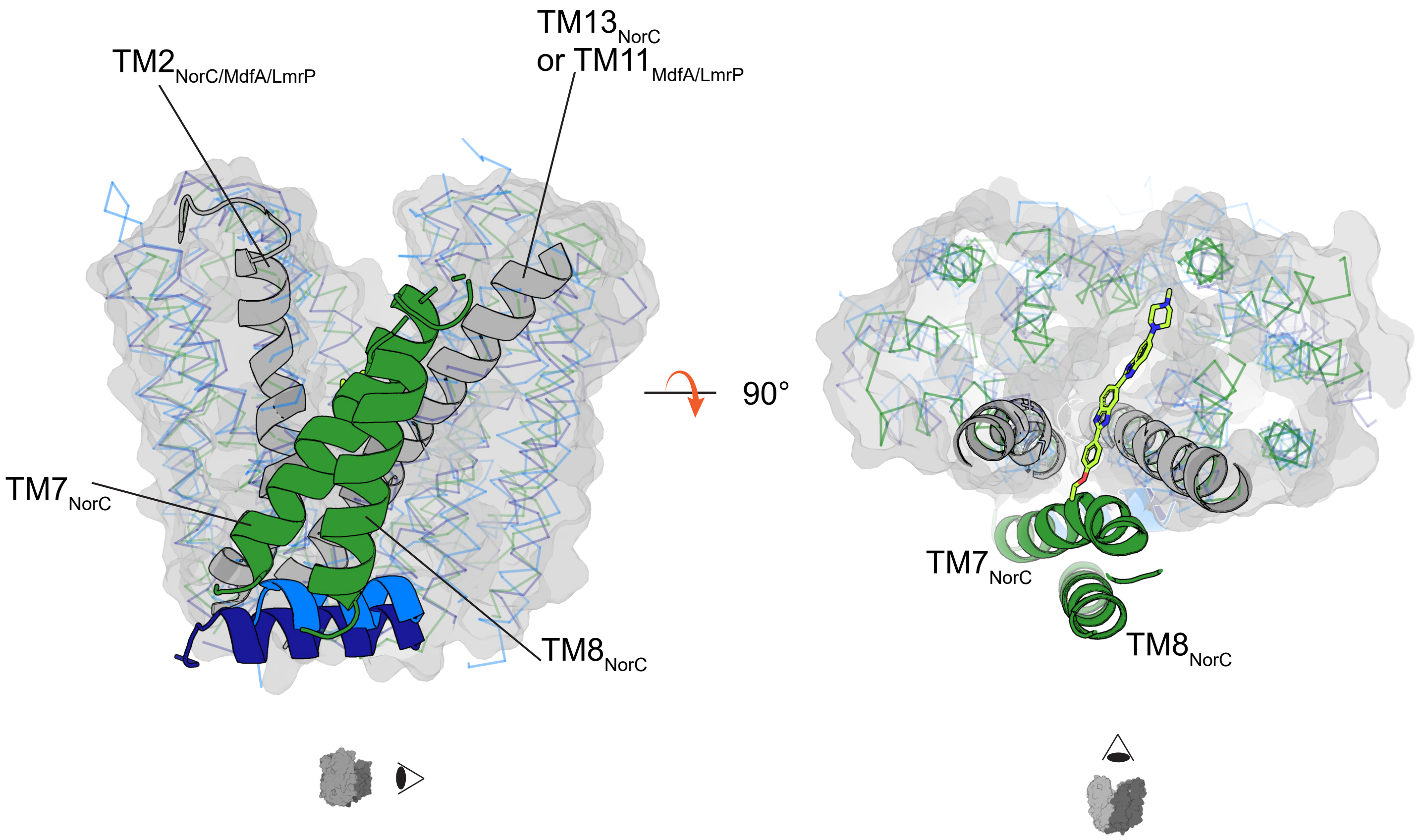

Supplementary Figure S6 | Superposition of NorC (green), MdfA (PDB ID: 6GV1, dark blue), and LmrP (PDB ID: 6T1Z, light blue) structures *(left)*. TMs 7 and 8 of NorC occlude a wide opening going from the vestibule to the upper half of the membrane, through a crevice made by TMs 2 and 13 (grey helices). Top view *(right)* showing the substrate Hoechst (sticks , bound to LmrP) clashing with TM7 of NorC.

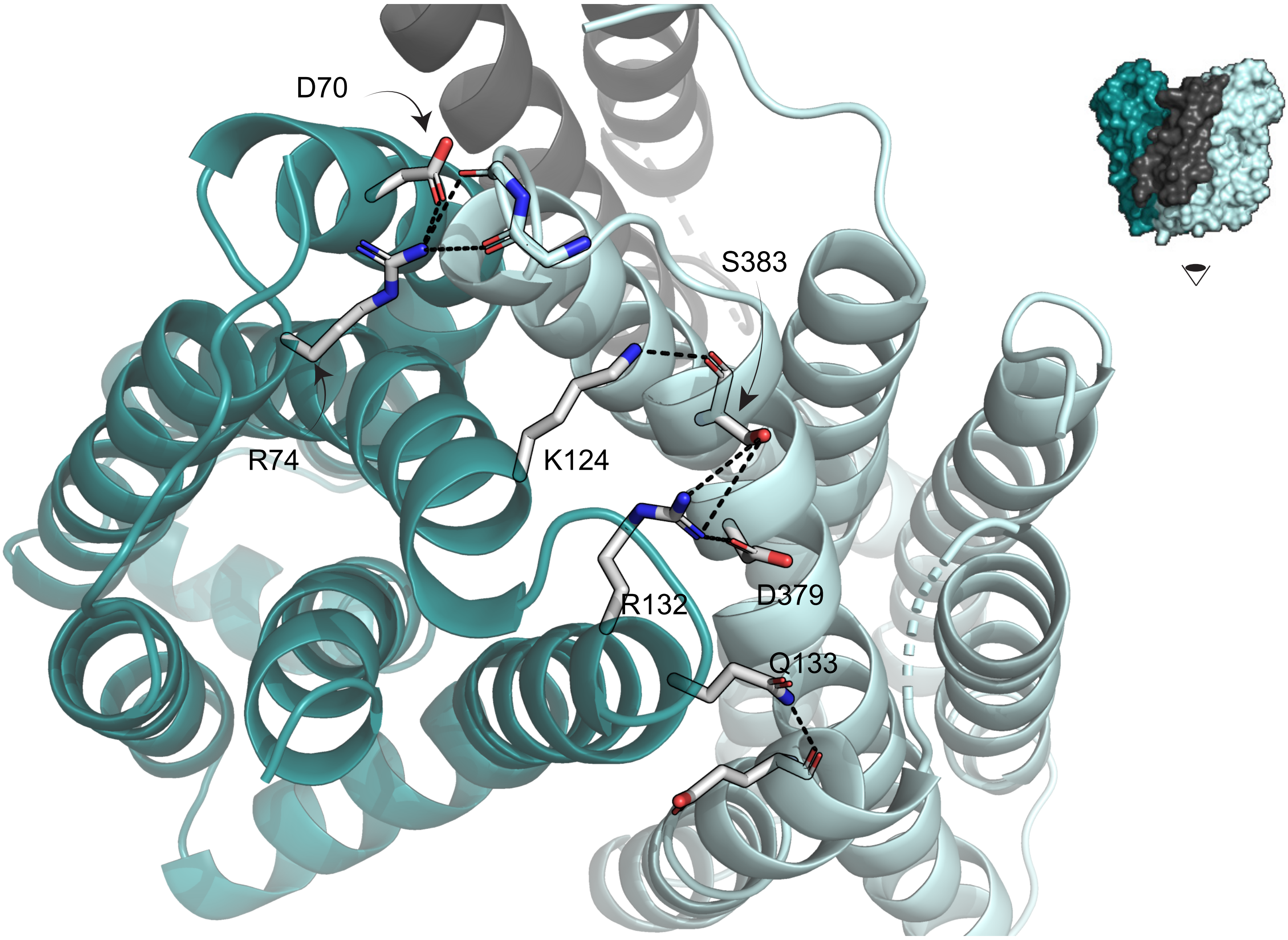

Supplementary Figure S7 | Polar interactions between two symmetry-related halves of NorC (N-terminal in teal, C-terminal in pale cyan) towards the cytosolic side.

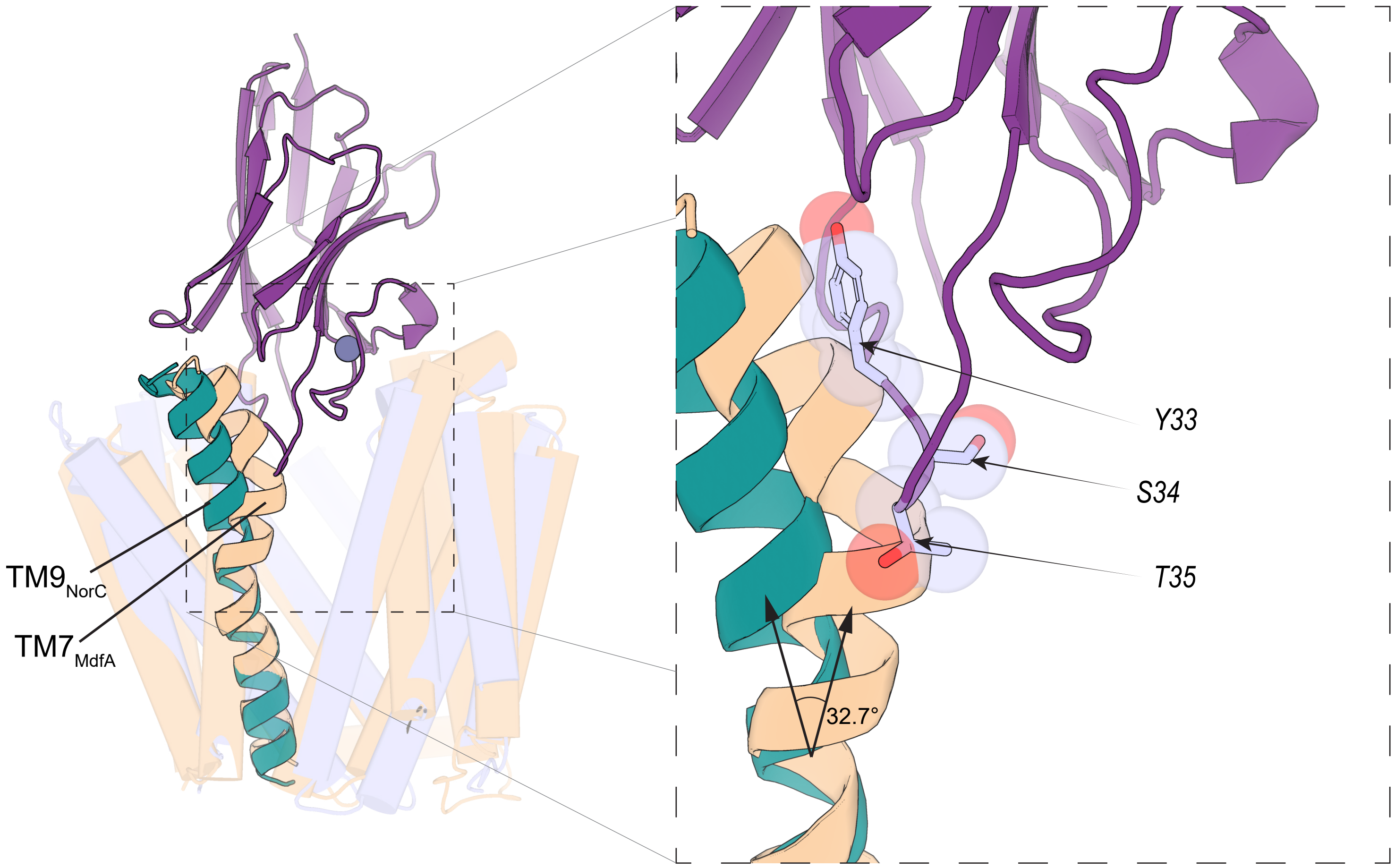

Supplementary Figure S8 | Superposition of NorC-ICab (light blue and purple) complex and MdfA (PDB ID: 6GV1, yellow). TM7 of MdfA and TM9 of NorC are shown in cartoon representation; where TM9 of NorC (teal) is relatively unbent whereas TM7 of MdfA kinks to an angle of 32.7° *(enlarged view, right)*. Side chains of ICab that would clash with TM7 of MdfA are shown in sticks and transparent spheres.

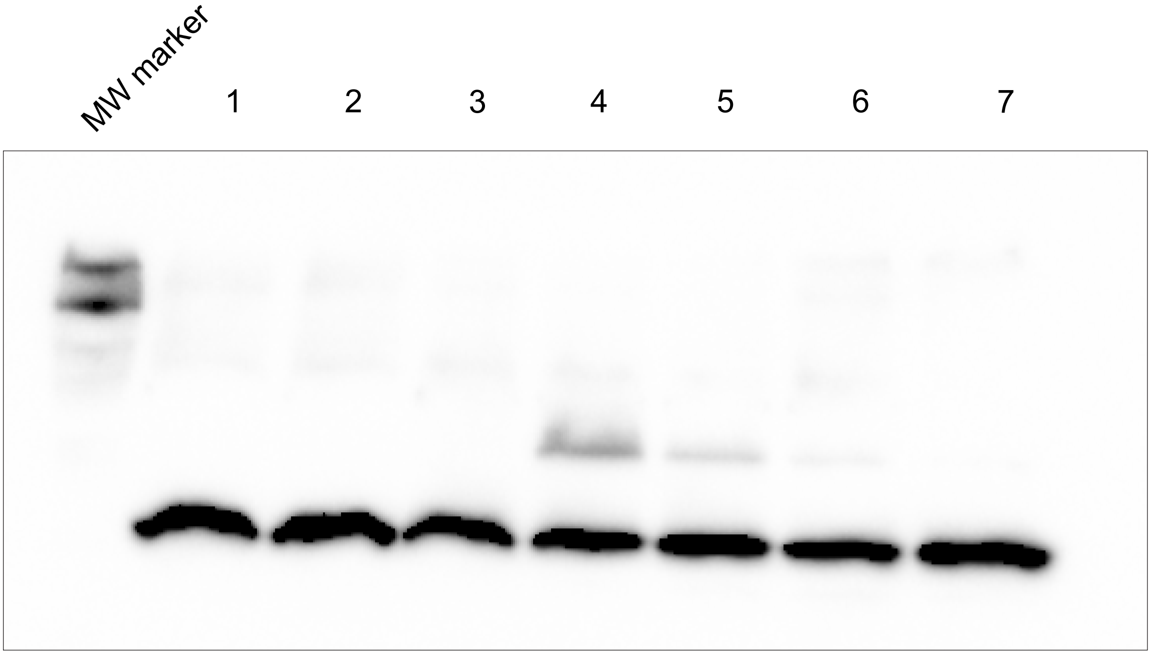

Supplementary Figure S9| Uncut western blot of PEG Maleimide accessibility assay shown in Figure 5b. Samples loaded in the lanes shown above are described as follows:

1: Membranes with NorC WT overexpressed

2. Membranes with NorC WT overexpressed + 1mM PEG maleimide
3. Membranes with NorC T378C overexpressed
4. Membranes with NorC T378C overexpressed + 1mM PEG maleimide
5. Membranes with NorC T378C overexpressed + 1mM TPP^+ + 1mM PEG Maleimide
6. Membranes with NorC T378C overexpressed + 60 μM ICab + 1mM PEG maleimide
7. Membranes with NorC T378C overexpressed + 1mM TPP^+ + 60 μM ICab + 1mM PEG maleimide.

The darker bands in the molecular wieght marker correspond to molecular masses of 75kDa and 100kDa.

	NorC WT vs ICab	NorC WT vs ICab with 1mM TPP (in both cell and syringe)
**Model**	One set of sites	One set of sites
**[Cell] (μM)**	20	20
**[Syr] (μM)**	200	200
**N (Sites)**	1.04 ± 0.005	1.14 ± 0.012
**K_d (nM)**	46.6 ± 8.90	35.6 ± 16.3
**ΔH (kcal/mol)**	-4.49 ± 0.063	-2.66 ± 0.082
**Offset (kcal/mol)**	0.119 ± 0.04	0.580 ± 0.06
**ΔG (kcal/mol)**	-10.0	-10.2

Supplementary Table ST1| Summary of ITC profiles shown in Figure 4c.
