# Supplementary material for: Structural basis of inhibition of a transporter from Staphylococcus aureus, NorC, through a single-domain camelid antibody
Source: Commun Biol. 2021 Jul 5;4:836. doi: 10.1038/s42003-021-02357-x (PMC8257674; doi:10.1038/s42003-021-02357-x)
Supplement: Supplementary file 6 — Reporting Summary [file 42003_2021_2357_MOESM6_ESM.pdf]

nature research

Corresponding author(s): Aravind Penmatsa

Last updated by author(s): 05/30/2021

Reporting Summary

Nature Research wishes to improve the reproducibility of the work that we publish. This form provides structure for consistency and transparency in reporting. For further information on Nature Research policies, see our Editorial Policies and the Editorial Policy Checklist.

Statistics

For all statistical analyses, confirm that the following items are present in the figure legend, table legend, main text, or Methods section.

n/a	Confirmed	
☐	☒	The exact sample size (*n*) for each experimental group/condition, given as a discrete number and unit of measurement
☐	☒	A statement on whether measurements were taken from distinct samples or whether the same sample was measured repeatedly
☐	☒	The statistical test(s) used AND whether they are one- or two-sided<br>*Only common tests should be described solely by name; describe more complex techniques in the Methods section.*
☒	☐	A description of all covariates tested
☒	☐	A description of any assumptions or corrections, such as tests of normality and adjustment for multiple comparisons
☐	☒	A full description of the statistical parameters including central tendency (e.g. means) or other basic estimates (e.g. regression coefficient) AND variation (e.g. standard deviation) or associated estimates of uncertainty (e.g. confidence intervals)
☒	☐	For null hypothesis testing, the test statistic (e.g. *F*, *t*, *r*) with confidence intervals, effect sizes, degrees of freedom and *P* value noted<br>*Give P values as exact values whenever suitable.*
☒	☐	For Bayesian analysis, information on the choice of priors and Markov chain Monte Carlo settings
☒	☐	For hierarchical and complex designs, identification of the appropriate level for tests and full reporting of outcomes
☒	☐	Estimates of effect sizes (e.g. Cohen's *d*, Pearson's *r*), indicating how they were calculated

Our web collection on statistics for biologists contains articles on many of the points above.

Software and code

Policy information about availability of computer code

Data collection	Malvern Microcal ITC software, Nanotemper PR.ThermControl DSF and MO.Control MST software, GE unicorn v7 FPLC software, MEGA X
Data analysis	XDS, CCP4i2, Phenix, Coot, Graphpad prism v5, Microsoft excel, MEGA X, PyMOL

For manuscripts utilizing custom algorithms or software that are central to the research but not yet described in published literature, software must be made available to editors and reviewers. We strongly encourage code deposition in a community repository (e.g. GitHub). See the Nature Research guidelines for submitting code & software for further information.

Data

Policy information about availability of data

All manuscripts must include a data availability statement. This statement should provide the following information, where applicable:

- Accession codes, unique identifiers, or web links for publicly available datasets
- A list of figures that have associated raw data
- A description of any restrictions on data availability

All accession codes of all deposited structures are mentioned in the article. All raw data associated with figures are provided with the script. There are no restrictions on data availability.
Raw data associated with the following figures are also provided in a separate file:
Figure 1a (and associated Supplementary Figure S1), Figure 3a-d, Figure 4b-c, Figure 5a-b, Suppl. Figure S2, Suppl. Figure S3a-b, Suppl. Figure S4a-d, Suppl. Suppl. table ST1.

Field-specific reporting

Please select the one below that is the best fit for your research. If you are not sure, read the appropriate sections before making your selection.

☒ Life sciences ☐ Behavioural & social sciences ☐ Ecological, evolutionary & environmental sciences

For a reference copy of the document with all sections, see nature.com/documents/nr-reporting-summary-flat.pdf

Life sciences study design

All studies must disclose on these points even when the disclosure is negative.

Sample size	Sample size determination was not applicable for any of the experiments as there was no experimental variability other than measurement variation. For DSF screens, n = 4 for independent replicates, for survival assays, n>3 for independent replicates, for MST, n=2-3 for every independent replicate, for ITC, n = 2 for independent replicates
Data exclusions	X-ray diffraction frames with higher R-merge were excluded to improved data quality. Some data points in DSF outlying 3 standard deviations were excluded.
Replication	All replications were successful. Raw data were normalized with values from control set, or the control set was subtracted from them, or were represented in percent change to achieve that.
Randomization	Not applicable in any of the experiments.
Blinding	No blinding was undertaken during experimental setup or analysis.

Reporting for specific materials, systems and methods

We require information from authors about some types of materials, experimental systems and methods used in many studies. Here, indicate whether each material, system or method listed is relevant to your study. If you are not sure if a list item applies to your research, read the appropriate section before selecting a response.

Materials & experimental systems

n/a	Involved in the study	
☐	☒	Antibodies
☒	☐	Eukaryotic cell lines
☒	☐	Palaeontology and archaeology
☒	☐	Animals and other organisms
☒	☐	Human research participants
☒	☐	Clinical data
☒	☐	Dual use research of concern

Methods

n/a	Involved in the study	
☒	☐	ChIP-seq
☒	☐	Flow cytometry
☒	☐	MRI-based neuroimaging

Antibodies

Antibodies used	Indian Camelid Antibody (ICab); 6x-His Tag Monoclonal Antibody (HIS.H8)(mouse origin)(invitrogen, Product # MA1-21315); Goat anti-Mouse IgG (H+L) Cross-Adsorbed Secondary Antibody, HRP (Invitrogen, Product # G-21040)
Validation	Citation for Indian Camelid Antibody from a previous publication:<br>"Kumar, S., Mahendran, I., Athreya, A., Ranjan, R. & Penmatsa, A. Isolation and structural characterization of a Zn(2+)-bound single-domain antibody against NorC, a putative multidrug efflux transporter in bacteria. J Biol Chem 295, 55-68 (2020)."<br>https://www.jbc.org/article/S0021-9258(17)49549-4/fulltext<br><br>For 6x-His Monoclonal Antibody; link for relative expression validation data from the manufacturer's website:<br>https://www.thermofisher.com/in/en/home/life-science/antibodies/invitrogen-antibody-validation/relative-expression-antibody-validation.html
