# Supplementary material for: Structural basis of inhibition of a transporter from Staphylococcus aureus, NorC, through a single-domain camelid antibody
Source: Commun Biol. 2021 Jul 5;4:836. doi: 10.1038/s42003-021-02357-x (PMC8257674; doi:10.1038/s42003-021-02357-x)
Supplement: Supplementary file 1 — Peer Review File [file 42003_2021_2357_MOESM1_ESM.pdf]

Description of Additional Supplementary Files

File name: Supplementary Data 1
Description: All raw data used to make the figures are provided in a single .xlsx file. The sheets have been renamed with the figure/supplementary figure panel it represents, and respective captions are provided there.
